# Cracking the Sulfur Code: Garlic Bioactive Molecules as Multi-Target Blueprints for Drug Discovery

**DOI:** 10.3390/ph18111766

**Published:** 2025-11-20

**Authors:** Faizul Azam, Md Jamir Anwar, Jordan Kahfi, Suliman A. Almahmoud, Abdul-Hamid Emwas

**Affiliations:** 1 Department of Medicinal Chemistry and Pharmacognosy, College of Pharmacy, Qassim University, Buraydah 51452, Saudi Arabia; s.almahmoud@qu.edu.sa; 2 Department of Pharmacology and Toxicology, College of Pharmacy, Qassim University, Buraydah 51452, Saudi Arabia; m.anwar@qu.edu.sa; 3 Division of Biological and Environmental Sciences and Engineering (BESE), King Abdullah University of Science and Technology (KAUST), Thuwal 23955-6900, Saudi Arabia; jordan.kahfi@kaust.edu.sa; 4 Core Lab of NMR, King Abdullah University of Science and Technology (KAUST), Thuwal 23955-6900, Saudi Arabia; abdelhamid.emwas@kaust.edu.sa

**Keywords:** organosulfur compounds, diallyl disulfide, pharmacological activity, molecular docking, liposomes, drug delivery

## Abstract

Garlic (*Allium sativum* L.) has served as a food source and medicinal agent for over thousands of years. Bioactive constituents, including allicin, diallyl sulfide/disulfide/trisulfide, ajoene, and *S*-allyl-cysteine, demonstrate antioxidant, anti-inflammatory, antithrombotic, antineoplastic, antimicrobial and neuroprotective properties. Convergent mechanistic evidence suggests the modulation of redox homeostasis, attenuation of pro-inflammatory signaling, regulation of platelet activation, and induction of apoptosis and cell-cycle arrest in tumor models. Computational studies, in conjunction with wet-lab data, offer molecular-level insights and guide candidate prioritization. Density functional theory elucidates radical-scavenging pathways and electronic descriptors that account for redox activity. Structure-based methods, including docking, molecular dynamics, and MM-GBSA, elucidate potential interactions between organosulfur scaffolds and enzymes or receptors pertinent to pharmacological effects. *In silico* ADME/Tox platforms predict generally favorable oral absorption for hydrophobic allyl sulfides, while polar derivatives exhibit more limited brain penetration. Emerging AI/ML pipelines combine network pharmacology with QSAR to focus on important targets and chemical types, while also spotting potential development. Formulation strategies, including nanoencapsulation and controlled-release systems, are utilized to stabilize labile thiosulfinates and modulate hydrogen-sulfide-releasing profiles, with potential applications in various disease conditions. Significant challenges encompass the standardization of preparations, variability in pharmacokinetics, heterogeneity in dose–response relationships, and interactions between drugs and nutrients or other drugs. The integration of mechanistic, computational, and formulation insights delineates a systematic approach to progress garlic-derived agents from diverse natural products to reproducible, mechanism-guided pharmaceuticals.

## 1. Introduction

Garlic (*Allium sativum* L.) is one of the oldest medicinal plants known to humans and has served as both a culinary essential and a therapeutic agent, spanning many civilizations, from ancient Egypt to modern pharmaceutical laboratories. The bioactivity of garlic is primarily attributed to various organosulfur compounds (OSCs), such as reactive thiosulfinates like allicin, lipid-soluble allyl sulfides and polysulfides (e.g., diallyl sulfide, diallyl disulfide, diallyl trisulfide), and water-soluble derivatives present in aged garlic extract (AGE), notably *S*-allyl-*L*-cysteine (SAC) and *S*-allyl-mercaptocysteine (SAMC). These compounds exhibit antimicrobial, antihypertensive, antithrombotic, anticancer, antidiabetic, and neuroprotective properties [1,2,3,4].

**Figure 1:**
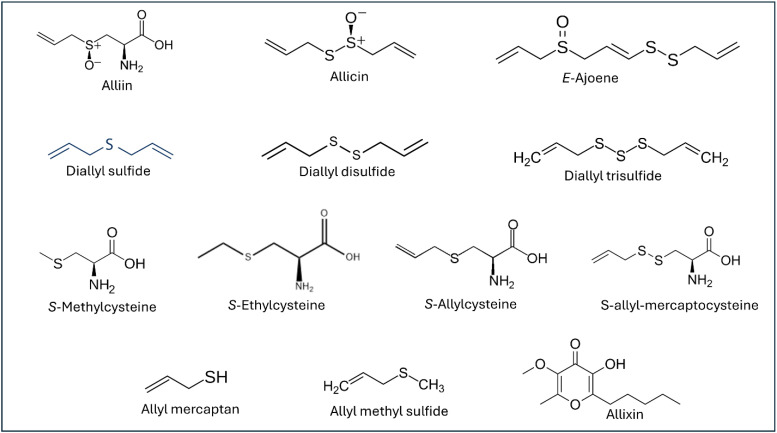
Chemical structures of the organosulfur compounds of garlic.

The modern pharmaceutical interest in garlic increased significantly after Cavallito and Bailey identified and isolated allicin (diallyl thiosulfinate) in 1944. They demonstrated the potent antimicrobial properties of this OSC and established the initial structure-activity relationships for bioactive constituents [5]. Subsequent decades of research have revealed several OSCs (Figure 1), each contributing unique therapeutic properties through sophisticated biochemical pathways involving enzyme inhibition, gene expression modulation, and cellular signaling pathway regulation [6,7,8,9].

Clinical and experimental findings demonstrate consistency for standardized AGE preparations, utilizing water-soluble compounds like SAC, providing enhanced stability and predictable effects. A recent meta-analysis of randomized trials involving hypertensive patients reveals modest yet significant reductions in blood pressure, especially at elevated daily doses [10,11]. Moreover, several studies indicates that garlic may contribute to the prevention and adjunctive management of chronic diseases, such as dyslipidemia, numerous cancers, type 2 diabetes, and infections [12,13,14,15,16,17,18,19]. However, the efficacy is significantly influenced by the processing and delivery methods of garlic. Variations such as raw cloves, dehydrated powders, oils, AGE, and black garlic exhibit distinct chemical profiles, compound stability, and bioavailability, resulting in differing clinical outcomes and tolerability [20,21,22,23,24].

Computational studies increasingly improve wet-lab research by clarifying reactivity, target engagement, and polypharmacology. Density-functional theory has clarified the formation pathways and electrophilicity of allicin [25]. Molecular docking, dynamics, and network pharmacology have identified potential target networks in cardiometabolic, neurodegenerative, infectious, and oncologic contexts, supporting hypotheses related to the thiol-reactive chemistry and H_2_S signaling of allyl polysulfides [26]. *In silico* frameworks facilitate the prioritization of scaffolds and support experimental design, addressing the chemical lability and promiscuity associated with OSCs.

The significance of formulation science arises from the fact that the reactivity that facilitates bioactivity simultaneously undermines self-life and systemic dissemination. Encapsulation methods, including cyclodextrin inclusion complexes for odor masking and thermal stability enhancement, nanoemulsions for improved dispersion and antibiofilm efficacy, and liposomal systems for increased solubility and protection of volatile essential-oil polysulfides, are progressing [27,28,29,30,31]. Food and pharmaceutical-grade platforms are starting to align on viable delivery solutions. The efficacy of these technologies will likely influence the translation of promising mechanistic effects into consistent *in vivo* outcomes [32].

Even with a wealth of reviews, an integrated, current synthesis is required due to the increasing volume of primary research on garlic OSCs and the relative lack of coverage in computational studies and formulation science [11,33,34,35,36,37,38,39,40,41,42,43,44,45]. By unifying advances in clinical efficacy, safety, and translational strategy, we present a coherent framework that reflects the evolving literature and supports the evidence-based development of garlic-derived therapeutics.

## 2. Pharmacological Activities

### 2.1. Cardiovascular Health

#### 2.1.1. Blood Pressure Regulation

The role of garlic in regulating blood pressure is multifaceted, involving direct vascular effects, hormonal modulation, and alterations in endothelial function [46]. Evidence from randomized clinical trials and animal models indicates that its bioactive spectrum affects multiple pathways involved in hypertension management [47]. A fundamental mechanism includes the enhanced synthesis of nitric oxide (NO), a vasodilatory molecule produced by endothelial nitric oxide synthase (eNOS). OSCs, including allicin and derivatives from aged garlic extract, can increase NO availability by either upregulating eNOS expression or safeguarding NO from oxidative degradation [8]. Increased vasodilation leads to a decrease in peripheral resistance, resulting in lower systolic blood pressure (SBP) and diastolic blood pressure. In addition to its role in NO-related dilation, garlic influences the renin-angiotensin system by inhibiting angiotensin-converting enzyme (ACE). *In vitro* studies indicate that γ-glutamyl cysteines present in garlic inhibit ACE activity; however, the extent to which these compounds survive digestion and reach target tissues in an intact form is uncertain, and thus their contribution to *in vivo* ACE inhibition is yet to be elucidated [48,49]. Inhibiting the conversion of angiotensin I to angiotensin II, a strong vasoconstrictor, leads to a reduction in arterial pressure and a decrease in aldosterone-mediated fluid retention. Preclinical *in vivo* studies in hypertensive rat models indicate that combining alliin with pharmaceutical ACE inhibitors such as captopril enhances antihypertensive effects compared with monotherapy, without evidence of possible toxicity in these models; however, these findings derive from controlled animal experiments, and dedicated toxicological and clinical studies are still required to establish the safety of such combinations in humans [8].

Experimental findings demonstrate that hydrogen sulfide (H_2_S), produced from the metabolism of garlic polysulfides, enhances vascular relaxation through the hyperpolarization of smooth muscle [17,50]. H_2_S functions in conjunction with NO, providing dual gaseous signaling mechanisms that contribute to the maintenance of reduced blood pressure. Variants in enzymes such as cystathionine γ-lyase or deficiencies in cofactors like low vitamin B_12_ may influence individual responses to garlic interventions. Populations with sub-optimal vitamin B_12_ levels may show reduced H_2_S production from garlic consumption, which could account for the variability observed in clinical outcomes.

Meta-analyses indicate varying statistical significance for reductions in systolic and diastolic blood pressure, influenced by study design and dosage form. Pooled analyses indicate significant reductions in hypertensive subgroups, whereas other studies report non-significant changes compared to placebo [51]. This inconsistency may arise from differences in preparation methods: enzymatically processed garlic or aged extracts appear to be more effective than raw powder in specific protocols, likely due to optimized concentrations of stable OSCs such as *S*-allylcysteine, which offer sustained actions targeting the endothelium.

Increased aortic stiffness raises SBP and pulse pressure by elevating ventricular afterload; garlic’s prostaglandin-like properties may mitigate this stiffness through a reduction in peripheral resistance [48,52]. Decreased vascular tension correlates with enhanced subendocardial perfusion, an essential element in mitigating ischemic injury in hypertensive conditions. Animal research provides mechanistic insights: extracts from related Allium species, such as *A. hookeri*, have demonstrated antihypertensive activity via simultaneous ACE inhibition and increased NO generation [53]. The data indicate that structural similarities among OSCs across species provide consistent bioactivity pertinent to human applications. Compounds such as *S*-allyl cysteine (SAC) derivatives influence endothelial function by modulating cyclic GMP pathways downstream of NO signaling, thereby extending their role beyond mere vasodilation to a more precise regulation of vascular tone. In addition, garlic-derived proteins, including methionine–glycine–arginine (MGR) tripeptide, histidine–aspartate–cysteine–phenylalanine (HDCF) tetrapeptide, garlic protein hydrolyzed product by pepsin (GPHP-P), and garlic protein hydrolyzed product by trypsin (GPHP-T), have shown a beneficial impact on cardiovascular system through their ACE inhibitory activity and protective effects against endothelial dysfunction [54]. Moreover, bradykinin, a potent vasodilatory peptide, contributes to cardiovascular health by activating eNOS, which increases NO production, and by enhancing the production of the platelet-aggregation-inhibitory prostaglandin, prostacyclin, through B2 receptor activity. Consequently, enhancing bradykinin activity through the inhibition of its degradation may be essential for promoting beneficial cardiovascular functionality. Garlic-derived proteins and their pepsin- and trypsin-hydrolysates, including GPHP-P and GPHP-T, exhibit antioxidant and ACE-inhibitory activity while maintaining bradykinin activity in hypertensive rats [55].

**Figure 2:**
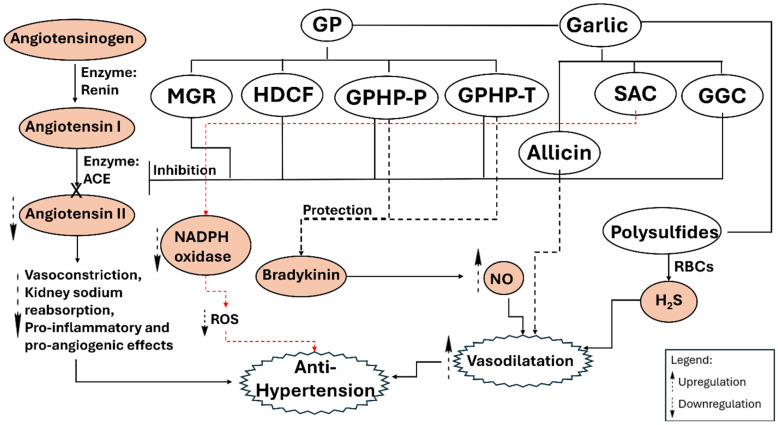
Garlic constituents protect the cardiovascular system through antihypertensive mechanisms, including ACE inhibition, downregulation of NADPH oxidase, and NO/H_2_S-driven vasodilation. GGC, gamma-glutamyl cysteine; GP, garlic peptides; GPHP-P, Garlic protein hydrolyzed product by pepsin; GPHP-T, Garlic protein hydrolyzed product by trypsin; HDCF, Histidine–Aspartate–Cysteine–Phenylalanine tetrapeptide; MGR, Methionine–Glycine–Arginine tripeptide; NO, nitric oxide; ROS, Reactive oxygen species; SAC, *S*-allyl-cysteine.

The variety of mechanisms, (Figure 2) including NO and H_2_S-mediated dilation, ACE inhibition, enhancement of aortic elasticity, and synergy with current pharmacotherapy, establishes garlic as a comprehensive approach to managing elevated blood pressure [8,17]. Biochemical individuality, influenced by genetic variation and nutrient status, suggests that a universal approach is improbable; personalized strategies informed by clinical profiling may enhance benefits and reduce risks. Future directions may involve computational modeling of organosulfur structure-activity relationships related to specific hypertensive phenotypes and the refinement of extraction technologies to achieve a balance between potency and tolerability.

#### 2.1.2. Lipid Profile Improvement

**Figure 3:**
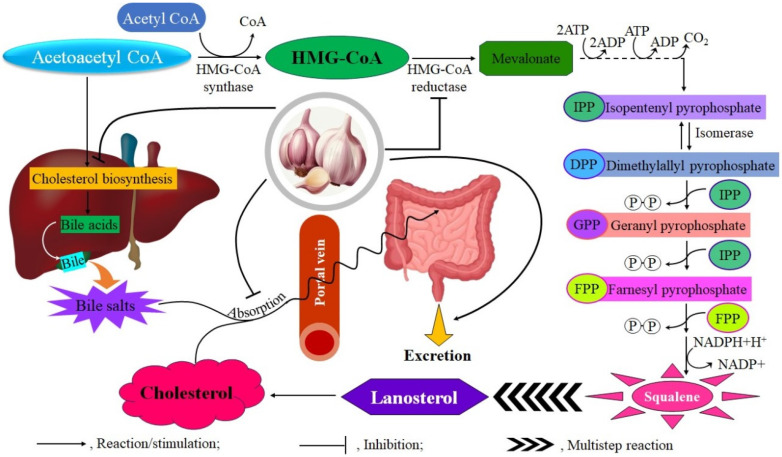
Garlic offers cardiovascular benefits by inhibiting HMG-CoA reductase, which reduces liver cholesterol biosynthesis, decreasing intestinal absorption, and enhancing elimination.

Compounds derived from garlic have gained significant attention in the context of cardiovascular diseases, especially concerning their ability to alter blood lipid profiles. The clinical evidence includes various study designs and garlic preparations, offering insights into the lipid-modulating effects of this commonly used botanical supplement. The lipid-lowering effects of garlic are largely attributed to OSCs that affect hepatic lipid metabolism by inhibiting key enzymes involved in cholesterol and fatty acid biosynthesis, such as HMG-CoA reductase, fatty acid synthase, and acetyl-CoA carboxylase (Figure 3). Furthermore, these compounds may increase the fecal excretion of bile acids and neutral sterols [56,57,58,59,60].

The clinical research base predominantly comprises controlled trials utilizing diverse garlic formulations. Numerous studies investigated various garlic preparations, such as dried garlic powder tablets, aged garlic extract, steam-distilled garlic oil, and different standardized extracts. Treatment durations varied from 12 weeks to 10 months, with sample sizes ranging from 23 to 115 participants. The evidence indicates generally positive effects on total cholesterol across various preparations. Quantitative reductions varied between 5.7% and 11.5% in studies that offered specific effect size estimates. Sobenin et al. observed an 11.5% reduction compared to placebo with the administration of time-released garlic powder tablets over a 12-week period [61]. Jain et al. reported a 5.7% reduction with the use of standardized garlic powder tablets [62]. A systematic review by Ackermann et al. indicated reductions of 0.03–0.45 mmol/L at 1 month and 0.32–0.66 mmol/L at 3 months [63]. Nonetheless, not all research indicated significant effects, as Neil et al. reported no notable difference over a 6-month period [64], and Berthold et al. observed non-significant outcomes with steam-distilled garlic oil [65].

The impact on low-density lipoprotein (LDL) cholesterol is noteworthy, as multiple studies indicate substantial reductions. Quantitative studies reported LDL reductions of 11% and between 11.8% and 13.8%. Steiner et al. observed a decrease ranging from 4% to 4.6% [66]. Further research indicated notable reductions in LDL levels, though specific numerical values were not provided. In contrast, high-density lipoprotein (HDL) cholesterol exhibited increases in various studies, with Sobenin et al. documenting an 11.5% rise and several other investigations noting significant increases without providing quantitative specifics [61].

Responses of triacylglycerol demonstrated variability across studies. Turner et al. reported a 12% reduction [67]. Numerous studies reported notable reductions but failed to present quantitative effect sizes, whereas Berthold et al. observed non-significant changes. This variability indicates that triacylglycerol responses may be less predictable or necessitate specific conditions to exhibit clinically significant changes [65].

Preparation-specific analyses indicate notable variations in efficacy profiles. Multiple studies utilizing garlic powder tablets indicated modest reductions in total and LDL cholesterol, along with some evidence of increases in HDL levels [67]. The time-released formulation investigated by Sobenin et al. is particularly noteworthy, demonstrating significant improvements in all three major lipid fractions. Standardized extracts, such as aged garlic extract and ethyl acetate preparations, demonstrated reductions in lipid parameters, though effect sizes remained generally modest and reporting consistency varied.

#### 2.1.3. Antithrombotic Effects

Garlic exhibits anti-thrombotic properties that serve as an important adjunct to its lipid-lowering and blood pressure-modulating effects, contributing to a comprehensive strategy for reducing cardiovascular risk. The plant’s ability to modulate platelet activation and aggregation is fundamental to its antithrombotic actions, as these processes are closely associated with thrombus formation and subsequent cardiovascular events, including myocardial infarction and ischemic stroke. Bioactive OSCs, specifically allicin, ajoene, and various diallyl polysulfides, have shown the ability to interfere with platelet aggregation pathways by disrupting the binding of fibrinogen to glycoprotein IIb/IIIa receptors on platelet surfaces [68,69]. This disruption diminishes platelet cross-linking in developing thrombi, consequently decreasing the risk of vessel occlusion. Ajoene, a compound derived from allicin and stable in specific processed forms of garlic, inhibits platelet aggregation by irreversibly modifying fibrinogen receptors and blocking ADP-induced activation [70].

**Figure 4:**
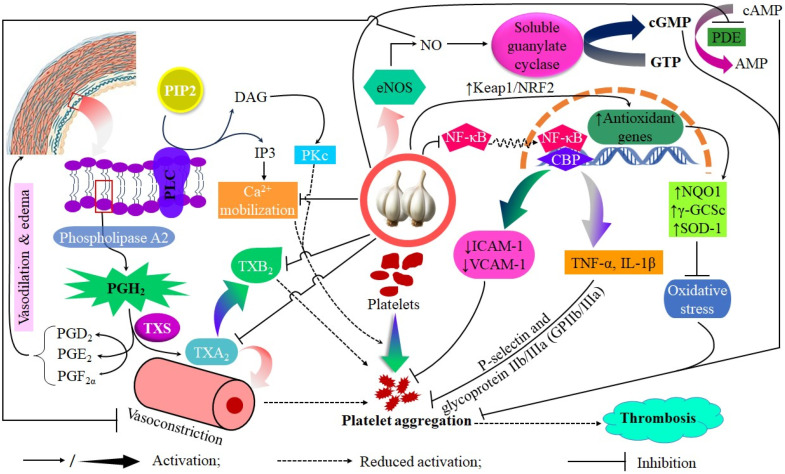
The proposed antiplatelet mechanisms of garlic include the inhibition of phospholipase A_2_ (PLA_2_), modulation of thromboxane A_2_ (TXA_2_) signaling resulting in reduced intracellular Ca^2+^ release and decreased TXB_2_ formation, attenuation of ADP- and collagen-induced platelet activation, diminished Ca^2+^ mobilization, and increased nitric oxide (NO) bioavailability.

*In vivo* evidence supports these biochemical findings. A double-blind, placebo-controlled trial assessed the effects of time-released garlic powder supplementation in patients with coronary heart disease over a 12-month period. The results indicated significant reductions in the calculated 10-year prognostic risk for acute myocardial infarction and sudden death, with a factor of 1.5 in men and 1.3 in women. These outcomes are likely associated with improvements in lipid profiles as well as reduced thrombotic tendencies [6,71]. Parallel reductions in platelet aggregation indices indicate a mechanistic relationship between garlic bioactives and decreased thrombus formation with chronic dietary inclusion. Platelets exhibit sensitivity to oxidative modifications that affect their reactivity threshold at the molecular level. The antioxidant properties of garlic, especially through SAC and phenolic compounds, reduce oxidative stress in circulating platelets by maintaining membrane fluidity and receptor functionality against ROS-induced changes [72]. The decrease in oxidative priming corresponds with diminished responsiveness to pro-aggregatory stimuli, including thromboxane A2 (TXA2). Additionally, allicin inhibits cyclooxygenase activity, thereby reducing TXA2 synthesis and limiting a crucial autocrine signal that facilitates platelet recruitment [73,74]. Moreover, garlic consumption has been linked to increased NO bioavailability, potentially enhancing platelet cGMP levels and reducing platelet adhesion to the endothelium [75]. Elevated cGMP levels result in decreased intracellular calcium concentration, which is essential for actin polymerization during the platelet shape change that occurs prior to aggregation [76]. Garlic offers a dual anti-thrombotic effect through chemical interference with receptor-mediated activation and modulation of endothelial-derived signals that promote an anti-clotting environment (Figure 4).

Studies utilizing animal models provide additional detail; ethanolic extracts of *Allium sativum* inhibit thrombus formation while preserving physiological hemostasis [70]. Ajoene and diallyl trisulfide demonstrate dose-dependent attenuation profiles, with the former resulting in prolonged inhibition likely due to irreversible enzyme modification in activated platelets. The release of hydrogen sulfide (H_2_S) mediated by DATS may also provide vasodilatory support, which indirectly diminishes platelet activation induced by shear stress in stenosed vessels. Processing significantly influences potency. Raw garlic, high in allicin, provides immediate but temporary inhibition due to its instability. In contrast, aged garlic extracts depend on stable thiol derivatives such as SAC, which, while appearing less effective against primary aggregation, can have long-term modulatory effects through endothelial conditioning and modification of the oxidative environment [24]. Furthermore, black garlic preparations maintain certain anti-thrombotic properties; however, the mechanisms may transition to phenolic-mediated antioxidant suppression of pro-aggregatory reactive oxygen species rather than direct receptor blockade [68,77,78,79]. These shifts may provide more effective long-term prevention strategies with lower bleeding risk profiles than the consumption of raw garlic in bolus form.

The dosing strategy determines whether antithrombotic responses remain within preventive limits or progress to clinically significant anticoagulation. Low to moderate intakes are likely to confer vascular protection with minimal hemorrhagic risk, whereas higher doses or concentrated supplements may enter pharmacological levels, requiring monitoring similar to that of conventional antithrombotics. Tailored strategies that account for initial clotting status, co-medication profiles, and specific cardiovascular outcomes may enhance this equilibrium.

### 2.2. Cancer Prevention

**Figure 5:**
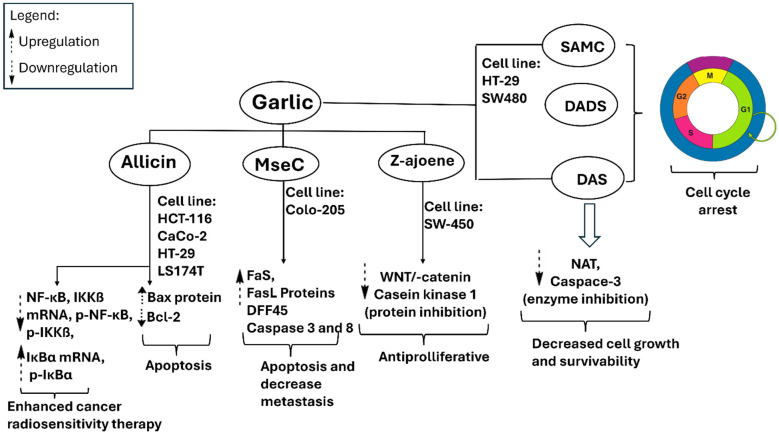
Metabolites derived from garlic have been evaluated in various cancer cell lines, demonstrating the ability to upregulate Bax and Fas/FasL while downregulating Wnt/β-catenin signaling and casein kinase 1α. Additionally, they inhibit NAT and caspase-3. These effects collectively inhibit cancer cells by enhancing apoptosis, restricting proliferation, diminishing cellular resistance, and augmenting radiosensitivity. DAS, diallyl sulfide; DADS, diallyl disulfide; SAMC, *S*-allyl-mercapto cysteine; MseC, methyl-*L*-selenocysteine.

Garlic has been identified as a potential natural agent for cancer prevention and treatment. Epidemiological studies often indicate a negative correlation between increased garlic consumption and the occurrence of various cancers [80,81,82,83]. The effects are primarily attributed to the organosulfur constituents of garlic, including allicin, DADS, DATS, SAC, and ajoene, which modulate essential oncogenic pathways. Proposed mechanisms encompass the induction of apoptosis, enforcement of cell-cycle arrest, inhibition of angiogenesis, and suppression of metastatic dissemination [84,85,86]. Moreover, the administration of OSCs in experimental animals decreases lipid peroxidation, suggesting a reduction in oxidative injury [87]. This process simultaneously increases tissue glutathione (GSH) levels and activates stress-responsive MAPK signaling pathways, specifically p38 and JNK [88,89]. Simultaneously, it downregulates pro-survival pathways, as indicated by decreased phosphorylated Akt (p-Akt) levels and reduced Bcl-2 expression [90,91]. These effects collectively enhance apoptotic competence and inhibit tumor initiation and progression *in vivo*. Figure 5 depicts the mechanistic framework of OSCs in cells together with outcomes from animal models that substantiate these pathways.

#### 2.2.1. Breast Cancer

**Figure 6:**
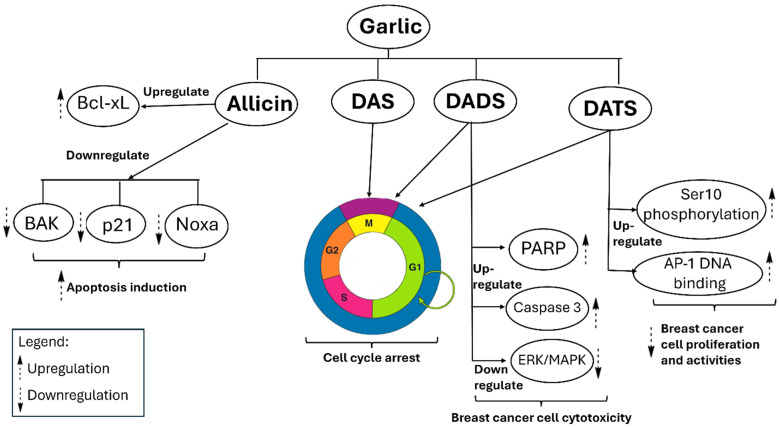
Proposed mechanisms of garlic compounds against breast cancer cells: induction of apoptosis, cell-cycle arrest, cytotoxicity, and suppression of proliferation.

Garlic extracts exhibit significant growth-inhibitory effects on breast cancer cells via various cellular pathways. Secondary metabolites, such as allicin, DATS, DADS, and DAS, exhibit a significant inhibitory effect on the proliferation of breast cancer cells, including human MCF-7. Except for allicin, these compounds inhibit cell division at the G0/M phases of the cell cycle, while allicin decreases glutathione (GSH) production [92]. Allicin demonstrates significant activity against the HCC-70 breast cancer cell line *in vitro*; however, it also shows toxicity to normal cells at elevated concentrations. Allicin functions by downregulating anti-apoptotic proteins such as Bcl-xL and upregulating pro-apoptotic proteins including p21, Bak, and Noxa. This process activates caspase-3 and caspase-8, resulting in apoptosis [93]. Furthermore, DADS enhances apoptotic signaling by elevating PARP cleavage and caspase-3 activity, while simultaneously decreasing TNF-α, a pro-inflammatory cytokine and breast cancer marker, via ERK/MAPK pathways. DATS enhances Ser10 phosphorylation of histone H3 *in vivo*, a mechanism linked to reduced proliferation of breast cancer cells [94]. DATS enhances AP-1 DNA-binding activity at the transcriptional level [95,96]. AP-1 regulates genes that control cancer cell behaviors such as proliferation, metastasis, and drug resistance [97]. Figure 6 illustrates the anticipated anticancer mechanisms associated with garlic.

#### 2.2.2. Colorectal Carcinoma

Laboratory studies consistently show significant anticolorectal cancer effects of garlic compounds. Aged garlic extract markedly reduces colon carcinogenesis induced by 1,2-dimethylhydrazine in animal models, leading to decreased adenoma formation and suppression of dysplastic lesion progression. The mechanism entails a delay in the cell cycle at the G2/M phase via the downregulation of cyclin B1 and CDK1, rather than through direct induction of apoptosis or cell cycle arrest. Compounds in garlic exhibit protective effects against the formation of aberrant crypt foci and demonstrate greater efficacy in preventing high-grade dysplastic adenomas relative to mild or moderate dysplasia [98].

**Figure 7:**
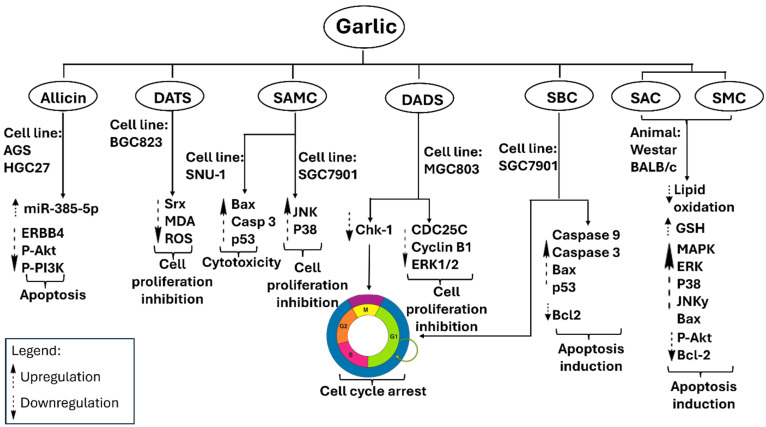
Metabolites derived from garlic inhibit cancer cells through mechanisms such as apoptosis, antiproliferative activity, cytotoxicity, and cell-cycle arrest, as demonstrated by multiparametric studies.

Fleischauer et al. provided a thorough summary of data regarding garlic-derived compounds, indicating significant reductions in colorectal cancer risk factors [99]. Figure 7 illustrates the proposed mechanisms through which garlic constituents inhibit colorectal carcinoma. Bat-Chen and colleagues assessed the effects of allicin on various colorectal cancer cell lines and determined that it promotes apoptosis by increasing Bax levels and enabling cytochrome c release into the cytoplasm while simultaneously reducing B-cell lymphoma-2 (Bcl-2) expression [100]. The simultaneous elevation of Bax and reduction in Bcl-2 serves as a well-established marker of apoptotic activity in colorectal cancer cells [101]. Huang et al. demonstrated that the combination of allicin and X-ray radiotherapy enhances the radiosensitivity of HCT-116 cells by downregulating NF-κB, IKKβ mRNA, p-NF-κB, and p-IKKβ, while upregulating IκBα mRNA and p-IκBα [102]. The modulation of the NF-κB pathway holds clinical significance, as its activity is associated with therapeutic resistance [103].

Z-ajoene exhibits significant antiproliferative effects in SW480 colon cancer cells by inhibiting Ser45 phosphorylation of β-catenin, a Wnt pathway effector modulated by casein kinase 1α (CK1α), and by decreasing the expression of c-Myc and cyclin B1. SAMC, DADS, and DAS exhibit significant antiproliferative activity. SAMC and DADS promote apoptosis in human colon cancer cells, such as HT-29 and SW480, by halting the cell cycle at the G2/M phase and enhancing caspase-3 activity [104]. In contrast, DAS demonstrates anticancer properties through the inhibition of arylamine N-acetyltransferase (NAT). NAT plays a role in carcinogenesis by activating procarcinogens and influencing tumor cell growth and survival [105].

Epidemiological research on colorectal cancer reveals discrepancies between findings from case–control studies and prospective cohort studies. Meta-analyses of case–control studies indicate a risk reduction linked to high garlic consumption. In contrast, prospective cohort studies reveal no significant association [106,107]. A recent meta-analysis concentrating solely on high-quality prospective studies revealed no significant protective association and unexpectedly indicated an increase in risk, although this finding was not statistically significant. The observed discrepancies may indicate variations in garlic preparation methods, consumption patterns, or methodological differences across study designs [108].

#### 2.2.3. Pancreatic Carcinoma

OSCs have been investigated for their anticancer properties concerning pancreatic ductal adenocarcinoma (PDAC) [109]. Evidence from epidemiological studies, preclinical models of PDAC, and the biology of infection and inflammation indicates that garlic and its derivatives operate through several mechanisms: (i) direct modulation of apoptosis, cell-cycle regulation, and oncogenic signaling in PDAC cells; (ii) enhancement of chemosensitivity and reduction in toxicity; and (iii) inhibition of tumor-associated microbes and periodontal inflammation, which are mechanistically linked to the initiation and progression of PDAC [110]. Observational studies indicate that increased consumption of garlic and onions is associated with reduced risk of pancreatic cancer [111]. A small, randomized trial involving patients with advanced gastrointestinal cancers, including pancreatic cancer, demonstrated that AGE supplementation preserved both the number and activity of natural killer cells [112]. However, comprehensive clinical trials focused on PDAC are necessary to determine effective formulations, dosing, pharmacokinetics, and safety, and to evaluate the impact of microbiome-targeted advantages on survival outcomes [44].

#### 2.2.4. Lung Cancer

Consumption of raw garlic exhibits significant protective effects against lung cancer, as evidenced by population-based studies. Case–control studies across various Chinese populations indicate a consistent risk reduction of 44–50% in individuals who consume raw garlic two or more times weekly compared to non-consumers. A comprehensive study involving 1424 lung cancer cases and 4543 controls demonstrated a dose-dependent inverse relationship, with the highest consumption category exhibiting an adjusted odds ratio of 0.56. The protective effect is significant across various demographic subgroups and seems to be independent of smoking status, indicating that garlic may provide protection to both smokers and non-smokers [113,114].

The lung-protective effects of garlic may be attributed to the pulmonary excretion of volatile OSCs, which may offer direct protective contact with lung tissue. *In vitro* studies indicate that garlic OSCs significantly inhibit the proliferation of lung cancer cell lines, such as A-549 and Calu-1, via various mechanisms, including cell cycle arrest, induction of apoptosis, and inhibition of angiogenesis. Recent studies have identified garlic compounds as potential agents for reversing drug resistance in non-small cell lung cancer, with allicin showing the capability to overcome Taxol resistance via mechanisms of cell cycle regulation [113,114,115].

### 2.3. Antimicrobial Activity

OSCs derived from garlic demonstrate extensive antimicrobial properties. Allicin exhibits bactericidal and fungicidal properties against both Gram-positive and Gram-negative bacteria, as well as *Candida* spp. Its primary mechanism of action involves S-thioallylation of cysteine residues in glutathione and various protein targets, such as thioredoxin reductase, alcohol dehydrogenase, and RNA polymerase, which disrupts redox homeostasis and essential metabolic processes [7,116]. Both *in vitro* and *in vivo* studies further elucidate these findings. Allicin and DATS demonstrate inhibitory effects on *Helicobacter pylori*, whereas diallyl sulfides exhibit suppression of methicillin-resistant *Staphylococcus aureus* (MRSA) in both models and clinical isolates. The potency of these compounds typically increases with the length of the sulfur chain, with the following order: diallyl tetrasulfide > DATS > DADS > DAS [117,118,119]. In addition to direct lethality, garlic components reduce pathogenicity: the vinyl disulfide ajoene acts as a quorum-sensing inhibitor in *Pseudomonas aeruginosa*, leading to the downregulation of virulence genes and disruption of biofilms; allicin exhibits antibiofilm properties and may enhance the efficacy of conventional treatments in certain scenarios [120,121]. Allicin inhibits bacterial DNA gyrase and, in fungi, enhances oxidative damage when used with amphotericin B, demonstrating complementary pathways for potential therapeutic applications [121,122]. The precursor alliin and stable aged-garlic components like SAC provide antioxidant and immunomodulatory effects, whereas the majority of antimicrobial activity is due to allicin and the more hydrophobic allyl sulfides (DAS, DADS, DATS, and higher polysulfides) [123,124]. Allicin’s volatility facilitates gas-phase activity that influences respiratory pathogens; however, its chemical lability necessitates delivery strategies that produce or stabilize allicin at sites of infection [125,126]. Evidence indicates that allicin is the principal antimicrobial component in garlic, while DADS, DAS, and DATS offer additional bactericidal, antibiofilm, and anti-quorum-sensing properties that may function additively or synergistically with conventional antimicrobials against key pathogens [40].

### 2.4. Metabolic Disorders

OSCs derived from garlic influence key pathophysiological pathways associated with metabolic disorders, such as insulin resistance, dyslipidemia, hepatic steatosis, and mild to moderate inflammation. Clinical evidence indicates that garlic supplementation enhances glycemic control and lipid profiles in individuals with type 2 diabetes. Additionally, trials involving nonalcoholic fatty liver disease (NAFLD/MASLD) demonstrate reductions in steatosis, improvements in liver enzymes, and beneficial improvements in body mass index following garlic-powder treatments [127,128,129,130]. Allicin enhances glucose uptake by activating AMPK through cysteine persulfidation signaling and promotes thermogenic remodeling (beiging) of adipose tissue, indicating increased energy expenditure as a complementary mechanism [131]. Aged garlic extract increases adiponectin levels in humans and enhances vascular-metabolic parameters, supporting the critical function of adiponectin in insulin sensitivity. DATS enhances glycemic control in diabetic models and inhibits adipogenesis in 3T3-L1 cells by modulating lipogenesis and fatty-acid metabolism [132]. In contrast, DADS exhibits variable effects, reducing insulin resistance and hepatic fat in obese mice *in vivo*, while promoting adipogenesis under certain *in vitro* conditions, underscoring the importance of dosage, exposure, and cellular state [133,134,135]. Garlic constituents such as alliin, *S*-allyl cysteine, and SAMC reduce inflammation, modulate insulin-receptor/AMPK signaling, and affect gut microbiota. These effects are combined on the AMPK–SREBP-1c and adipokine pathways, which are associated with enhanced insulin sensitivity and lipid homeostasis [136,137,138,139,140]. Human trials and mechanistic studies indicate that allicin and allyl sulfides, enhanced by aged-garlic constituents, provide multifactorial benefits for metabolic syndrome components, NAFLD, and insulin resistance. However, there is notable heterogeneity in preparations, doses, and endpoints [141].

### 2.5. Neuroprotection

Garlic-derived OSCs demonstrate neuroprotective effects in both acute and chronic neurological injury models. These effects are mediated through the modulation of oxidative stress, neuroinflammation, proteostasis, and mitochondrial resilience. SAC consistently provides protection against ischemic and neurodegenerative insults among water-soluble constituents. It activates Nrf2-dependent antioxidant defenses, mitigates endoplasmic reticulum stress, reduces infarct burden and neurological deficits following experimental stroke, and interferes with amyloid-β aggregation while enhancing learning and memory in rodent models of Alzheimer’s disease [142,143,144].

Lipophilic allyl polysulfides exhibit significant anti-neuroinflammatory properties in microglia. In LPS-activated BV2 cells, DATS and DADS inhibit nitric oxide and pro-inflammatory cytokines through NF-κB suppression, with DATS demonstrating the highest activity. *In vivo*, DATS mitigates neuroinflammation and enhances recovery following traumatic brain injury via PGK1/Nrf2 signaling [145,146]. Allicin exhibits neuroprotective effects in cerebral ischemia–reperfusion, as well as in traumatic and hemorrhagic brain injuries, by reducing oxidative damage, inhibiting TLR4–NF-κB/MAPK pathways, maintaining blood–brain barrier integrity, and enhancing functional recovery [147,148,149]. Recent findings indicate that alliin, the stable precursor of allicin, may mitigate neuronal injury in preclinical models; however, its efficacy in microglial inflammation models appears to be less pronounced compared to DATS/DADS [146,150]. Aged garlic extract, which is rich in SAC and related hydrophiles, has been shown to reduce Aβ-induced cognitive impairment and neuroinflammation in rodent models, as well as reverse significant endotoxin-induced transcriptomic alterations in microglia [151,152]. Furthermore, the neuroprotective profile of allyl polysulfides, particularly DATS, is enhanced by their ability to donate hydrogen sulfide (H_2_S). This gasotransmitter activates Nrf2/HO-1, reduces microglial activation, and enhances mitochondrial function in models of neurodegeneration [153].

Garlic has been extensively investigated in pharmacological research, mostly due to its bioactive OSCs. A summary table of preclinical data is presented below, covering *in vitro*, *in vivo*, and *in silico* research, listing the main molecular targets and mechanisms, the model systems used, and notable results, together with relevant references (Table 1).

## 3. Formulations of Organosulfur Compounds

Several formulations of OSCs are aimed at enhancing aqueous dispersion and solubility, providing protection against hydrolysis, volatilization, and oxidation, reducing odor, and improving bioavailability and tissue targeting.

### 3.1. Nanoemulsions and High Internal Phase Emulsions (HIPEs)

Nanoemulsions significantly enhance the dispersion, stability, and pharmacological efficacy of highly volatile or chemically labile garlic actives by protecting against premature decomposition [191]. Recent studies demonstrate that allicin nanoemulsions work synergistically with ε-polylysine to inhibit both planktonic and biofilm forms of E. coli, exhibiting a low fractional inhibitory concentration (FIC) index [27]. Additionally, these nanoemulsions modulate cell surface charge, indicating that nanodroplets enhance agent-cell contact and efficacy. DADS has been nanoemulsified using soy-protein emulsifiers, resulting in effective droplet formation, enhanced physicochemical stability, and suitability for complex food systems [192]. HIPEs extend beyond conventional emulsions by physically entrapping allicin within a fiber-stabilized interface that resists coalescence and facilitates thermo-responsive release. This approach offers an alternative method for confining this thiosulfinate under conditions of thermal and oxidative stress [193]. Complementary studies indicate that garlic essential oil nanoemulsions, comprising a garlic-only mix of DAS/DADS/DATS/ajoene, exhibit broad-spectrum antibacterial and antibiofilm activity, along with enhanced kinetic stability compared to coarse dispersions. These findings endorse nanoemulsification as an effective formulation strategy for OSCs [194,195].

### 3.2. Liposomes

Phospholipid bilayers protect sulfur functionalities from hydrolysis and oxidation, facilitating sustained, cell-interactive delivery of specific garlic actives. A pivotal study on allicin nanoliposomes indicated the formation of approximately 145 nm vesicles exhibiting a negative ζ-potential of −40 mV and sustained *in vitro* release [196]. This study demonstrates the efficient entrapment and controlled release of a single, labile thiosulfinate. Recent studies indicate that DADS co-loaded with cisplatin into nanoliposomes enhances cytotoxicity compared to either free drug in breast and lung cancer cell lines [197], suggesting that allyl disulfides may function as synergistic payloads within clinically established vesicles. Furthermore, PEGylated liposomes containing DATS improved doxorubicin chemosensitization, demonstrating high entrapment efficiency and significant antitumor activity in a colorectal cancer model [198]. In addition, liposomal DADS enhanced *in vitro* activity and was investigated in conjunction with oxaliplatin [199]. Supporting evidence for liposomal cisplatin platforms and recent reviews of liposome technology highlight the stability, adjustable release, and membrane fusion benefits relevant to allicin/DADS/DATS formulations [200,201,202,203].

### 3.3. Solid Lipid Nanoparticles and Related Lipid Carriers

Solid lipid nanoparticles (SLNs) provide solid-state matrices that reduce diffusion, limit volatility, and protect reactive sulfur moieties. Allicin-loaded solid lipid nanoparticles (SLN) functionalized with folic acid and chitosan demonstrated high encapsulation efficiency, a size range of approximately 80–90 nm, and enhanced *in vitro* anticancer activity compared to free allicin. This indicates that lipid solids can effectively stabilize and deliver a labile thiosulfinate [204]. Folic-acid–decorated DATS-SLN effectively targeted triple-negative breast cancer cells, enhancing uptake and efficacy. This approach mitigated the issues of DATS hydrophobicity and short half-life while maintaining the integrity of the garlic-only payload [205]. Comprehensive evaluations of lipid nanoparticles in the delivery of natural products and cancer treatment highlight SLN/NLC platforms as viable methods for converting DADS/DATS/allicin into constructs that are either tumor-targeted or enhanced for bioavailability [206,207].

### 3.4. Polymeric Nanoparticles and pH-Responsive Matrices

Biopolymers enhance mucoadhesion and facilitate site-selective release of polar garlic compounds. SAC, a water-soluble amino acid derived from garlic, has been encapsulated in chitosan nanoparticles for intranasal administration. This formulation enhances brain bioavailability in ischemia models, demonstrating that both lipophilic allyl sulfides and ionic garlic compounds can benefit from nanoscale carriers [208]. The allicin precursor, alliin, is encapsulated in pH-responsive gel beads that protect against gastric acidity, facilitating prolonged intestinal release. This mechanism aligns the exposure window with the targeted intestinal biophase while minimizing acid-catalyzed decomposition [209]. Previous research on alliin/alliinase tablets and their release analytics supports these encapsulation strategies and highlights the formulation challenge of producing allicin in situ within physiological constraints [210].

### 3.5. Cyclodextrin Inclusion Complexes

The complexation of host and guest molecules with cyclodextrins diminishes odor, enhances apparent solubility, and stabilizes volatile sulfides. Allicin complexes with α- and β-cyclodextrin effectively mask odor and stabilize the thiosulfinate. A recent study on β-cyclodextrin achieved approximately 50% drug loading with around 86% entrapment, enhancing *in vitro* performance and addressing allicin’s instability through host-guest interactions [30,211]. A study conducted by Qian et al. focused on encapsulating DADS and garlic oil within β-cyclodextrin to reduce soil interactions and minimize oxidation, degradation, and evaporative loss of sulfur volatiles [212]. Numerous studies on garlic-oil/β-CD inclusion, involving mixtures of DAS, DADS, DATS, and ajoene, demonstrate enhanced chemical stability, water compatibility, and odor masking, thereby validating CDs as effective scalable carriers for garlic bioactive compounds [213,214].

### 3.6. Hydrogels, Films, and Nanofibers

Soft matrices effectively localize garlic actives at moist interfaces, facilitating controlled release and enhancing tissue interaction [215]. A hyaluronic-acid methacryloyl hydrogel that delivers allicin-enhanced multiterritory perforator flap survival in rats and activates PI3K/AKT and HO-1/NRF2 signaling pathways, exhibiting a sustained and biocompatible release of a volatile thiosulfinate under *in vivo*-like conditions [216]. In addition, a collagen hydrogel incorporating allicin-derived Ag nanoparticles utilized allicin from garlic as the exclusive organic active/capping agent, demonstrating antimicrobial and wound-healing properties [217]. Furthermore, electrospun core-sheath fibers infused with garlic extract demonstrate the viability of nanofibrous dressings that retain exclusive garlic composition [215].

### 3.7. Ethosomes and Niosomes

Ethanol-enriched ethosomes and nonionic-surfactant niosomes enhance stratum corneum penetration and decrease volatility. Garlic-oil ethosomes exhibit notable, broad-spectrum antibacterial activity against clinical isolates and enhanced skin distribution relative to conventional carriers [218]. Additional studies suggest antidermatophytic and wound-healing capabilities associated with the garlic formulation [219]. Prototype niosomes encapsulating allicin, prepared via thin-film hydration, illustrate the cost-effectiveness of vesicular systems for thiosulfinates. Additionally, extensive reviews on niosomes emphasize carrier characteristics relevant to allicin, DAS, DADS, DATS, and ajoene [220,221].

## 4. Computational Insights

Recent advancements in computational chemistry have significantly enhanced our understanding of garlic bioactive compounds, especially organosulfur molecules like allicin, DAS, DADS, and DATS. The combination of molecular docking, quantum mechanical calculations, quantitative structure-activity relationships, molecular dynamics simulations, and artificial intelligence methodologies has significantly enhanced our comprehension of garlic’s bioactive components and their molecular mechanisms of action [198,199]. The small size, high polarizability, and soft Lewis basicity render them suitable ligands for metallo-enzymes and redox-sensitive sites, prompting significant computational studies [222].

### 4.1. Molecular Docking

Molecular docking functions at the intersection of structural biology and computational chemistry, with the objective of mathematically predicting the fitting of a ligand into a protein’s active site and its binding affinity. This process fundamentally models the spatial and energetic compatibility between a small molecule, typically a bioactive compound like allicin, diallyl disulfide, or alliin, and a macromolecular target, which may include an enzyme, receptor, or transporter. Interactions are evaluated using scoring functions that estimate free energy changes resulting from hydrogen bonding, hydrophobic contacts, van der Waals forces, electrostatic complementarity, and variations in conformational entropy.

Molecular docking studies have identified various biological targets for garlic OSCs. Molecular docking using AutoDock Vina yielded predicted binding scores ranging from −3.7 to −8.3 kcal/mol across different protein families, such as EGFR mutants T790M/C797S [223], bacterial topoisomerase IV [224], and ferroptosis-related proteins [225]. In the estrogen receptor-α docking study [226], alliin demonstrated the most favorable AutoDock Vina binding score of −4.8 kcal/mol compared to the other tested ligands.

**Figure 8:**
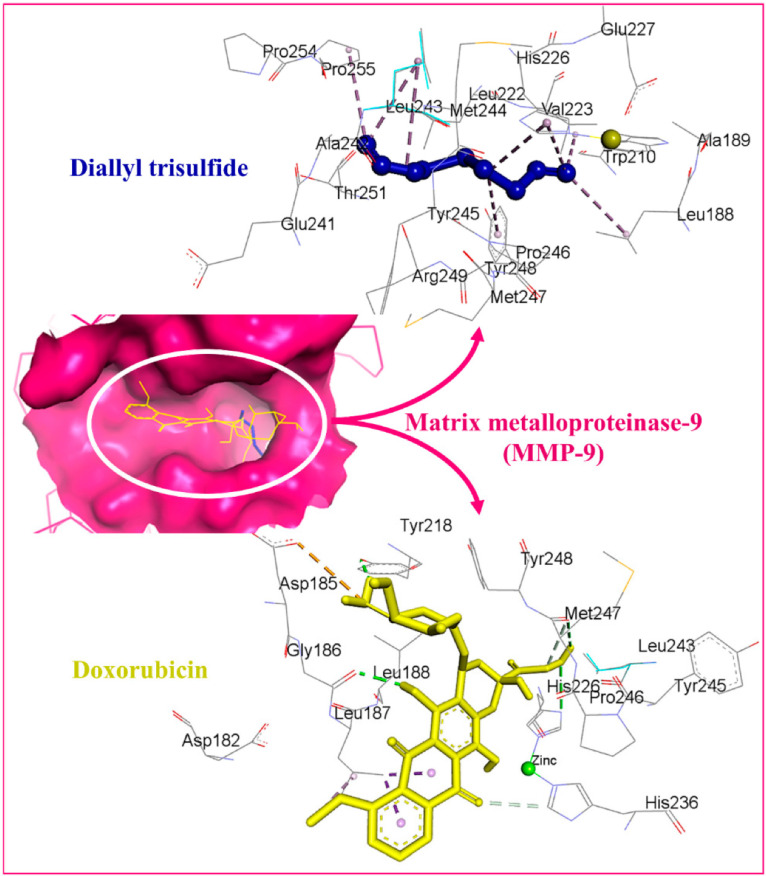
Docked DATS (blue) and doxorubicin (yellow) within the matrix metalloproteinase-9 (MMP-9) active site. The binding pocket is shown as a dark-pink surface, interacting residues as gray sticks, and intermolecular interactions as dashed lines. Adapted from Alrumaihi et al. [198].

**Figure 9:**
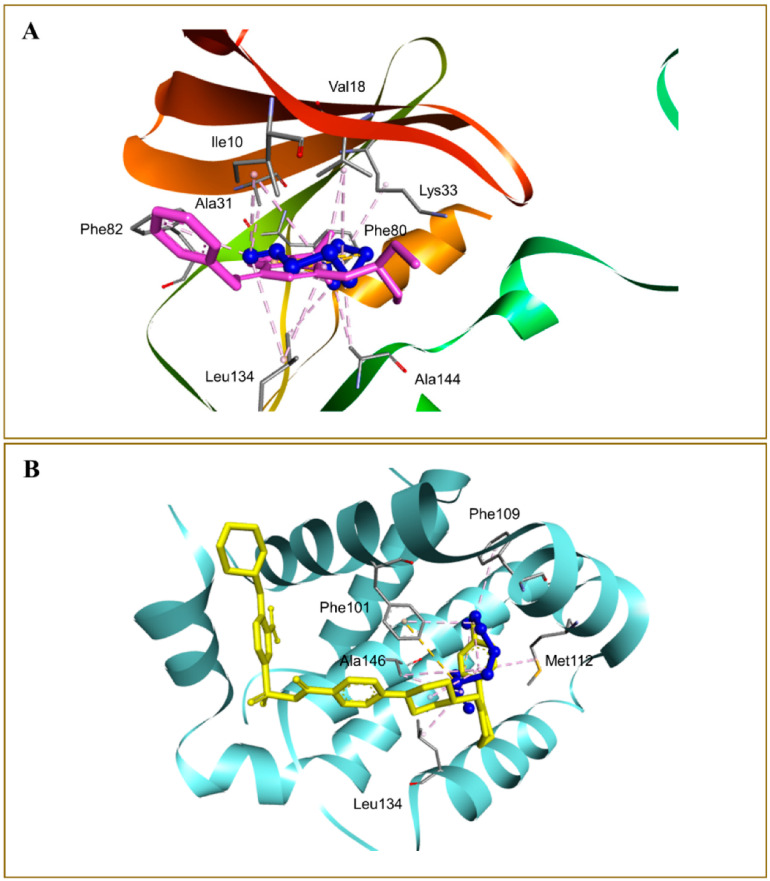
DATS (blue, ball-and-stick) is docked in the binding pockets of cyclin-dependent kinase 2 (**A**) and the apoptosis regulator Bcl-2 (**B**). Native co-crystallized ligands are represented as purple and yellow sticks. Nonbonded interactions are represented by dashed lines. Adapted from Alrumaihi et al. [198].

Alrumaihi et al. [198] utilized AutoDock Vina to assess the docking of diallyl trisulfide (DATS) in comparison to the reference drug doxorubicin (DOXO) across twelve potential anticancer targets. The docking results identified MMP-9 as the most favorable target for DATS, exhibiting a binding energy of −4.6 kcal/mol, while DOXO demonstrated a stronger binding affinity to the same protein at −8.9 kcal/mol. DATS exhibited significant affinities for CDK2 (−4.3 kcal/mol), Bcl-2 (−4.2 kcal/mol), and MMP-2 (−4.0 kcal/mol). In contrast, DOXO exhibited the strongest interaction with JAK2 (−9.6 kcal/mol), while DATS demonstrated only a moderate engagement with this protein (−3.5 kcal/mol). Figure 8 presents interaction maps for DATS with CDK2 and Bcl-2. DATS exclusively formed hydrophobic contacts with all macromolecular targets, whereas DOXO established both polar and non-polar interactions. The interacting residues for MMP-9 comprised L188, A189, W210, L222, V223, H226, E241, A242, L243, M244, Y245, P246, M247, Y248, R249, T251, P254, and P255 (Figure 9). DOXO, recognized as a DNA intercalator, exhibited moderate affinity for nucleic acid docking (−5.5 kcal/mol), while DATS demonstrated weak DNA binding (−2.1 kcal/mol).

A separate study conducted by the same group [199] investigated the effects of liposomal diallyl disulfide (DADS) and oxaliplatin (OXA), a standard drug, on the proliferation of colorectal cancer cells. DADS and OXA were docked to sixteen targets associated with anticancer drug discovery.

**Figure 10:**
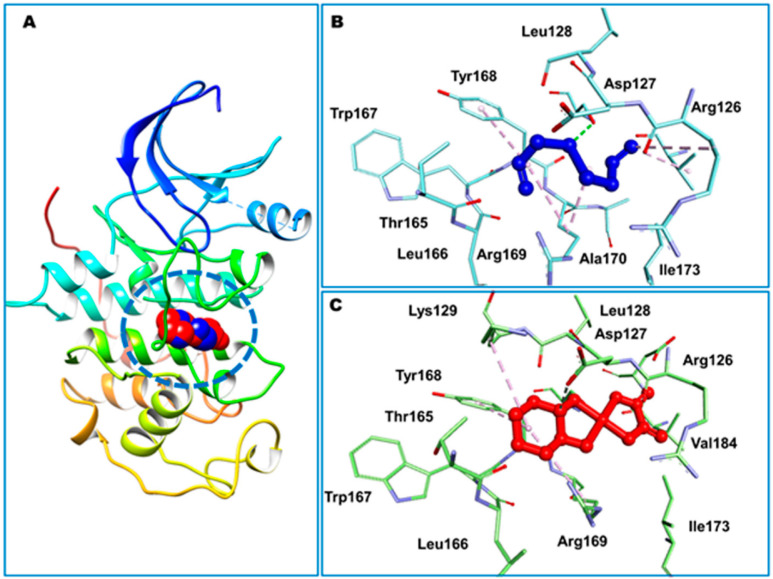
Docked DADS (blue) and OXA (red) within cyclin-dependent kinase 2 (CDK2; (**A**)). CDK2 is illustrated as ribbons, with ligands occupying the binding site represented in space-filling (CPK) format. The minimum-energy conformations of DADS (**B**) and OXA (**C**) are depicted using a ball-and-stick model, with interacting residues represented as sticks. Dashed lines represent intermolecular interactions. Adapted from Alrumaihi et al. [199].

**Figure 11:**
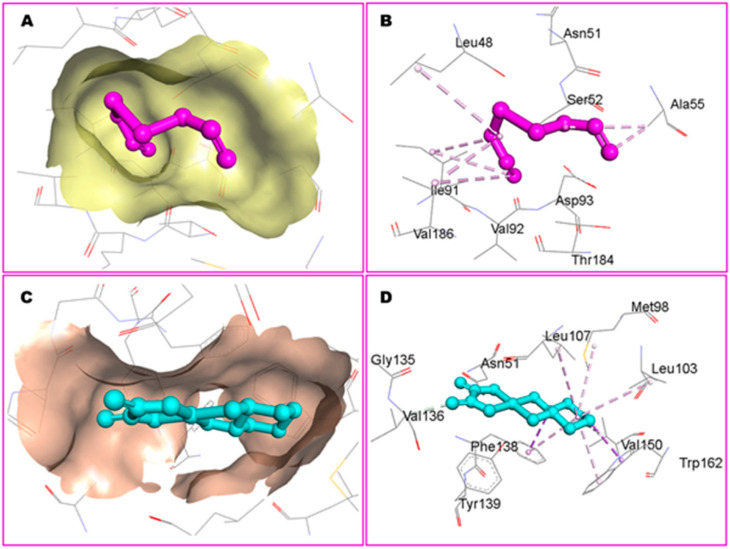
The binding site of heat shock protein 90 (Hsp90) is depicted as a molecular surface (**A**,**C**), which accommodates the docked DADS (purple) and OXA (cyan). Binding-site residues are illustrated using line representation (**B**,**D**). Adapted from Alrumaihi et al. [199].

DADS exhibited the highest affinity for CDK2 at −5.23 kcal/mol, while OXA demonstrated a weaker binding affinity of −2.11 kcal/mol for CDK2. In contrast, Hsp90 exhibited the highest binding affinity for OXA (−7.06 kcal/mol), whereas DADS showed a moderate affinity for Hsp90 (−3.84 kcal/mol). The compounds established both hydrophilic and hydrophobic interactions with the macromolecular targets. The CDK2 binding pocket, which includes R126, D127, L128, K129, T165, L166, W167, Y168, R169, I173, and V184, serves as a strong interface for DADS and OXA (Figure 10). In Hsp90, N51 formed a hydrogen bond with OXA, while DADS did not exhibit similar polar interactions. Non-polar contacts were associated with M98, L103, L107, G135, V136, F138, Y139, V150, and W162 (Figure 11).

**Figure 12:**
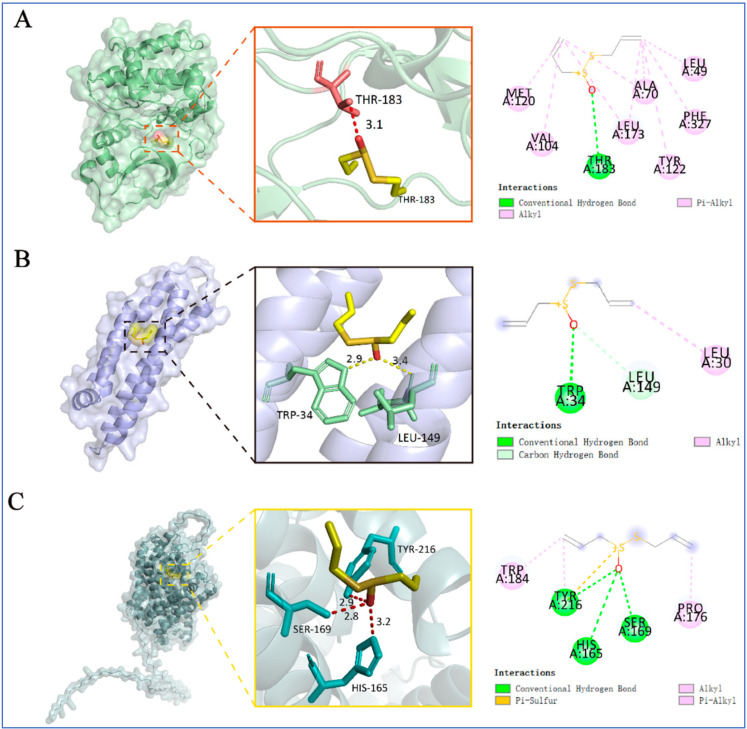
Molecular docking of allicin (ALC) with three targets. (**A**) ALC bound to protein kinase A. (**B**) ALC bound to the dopamine transporter. (**C**) ALC bound to apolipoprotein E. Adapted from Zeng et al. [26].

Furthermore, Zeng et al. [26] assessed the neuroprotective potential of allicin using molecular docking by the CB-DOCK2 program to predict binding poses and affinities for three targets: the dopamine transporter (DAT), protein kinase A (PKA), and apolipoprotein E (APOE). The binding energies predicted were −4.5 kcal·mol^−1^ for PKA, −3.5 kcal·mol^−1^ for DAT, and −3.4 kcal·mol^−1^ for APOE. Hydrogen bonds were detected in all complexes, suggesting favorable ligand–target interactions (Figure 12).

**Figure 13:**
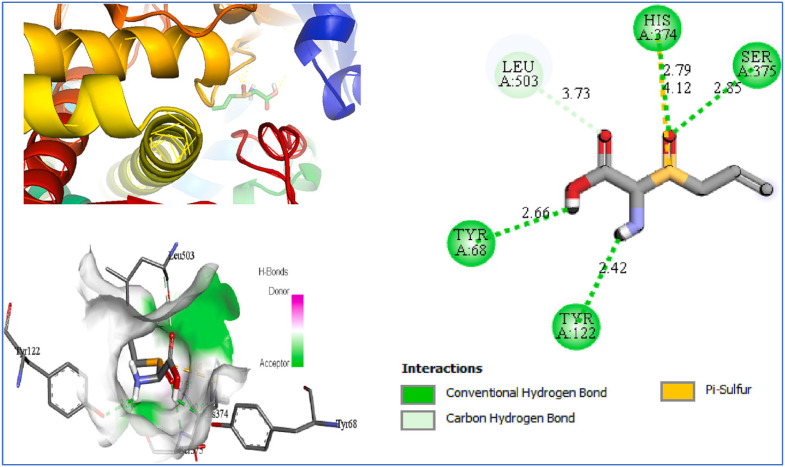
Alliin bound in the CYP51B active site pocket. Adapted from Bouamrane et al. [227].

Bouamrane et al. [227] employed molecular docking to evaluate garlic-derived compounds targeting Aspergillus fumigatus 14α-sterol demethylase (CYP51B). Alliin exhibited a predicted binding energy of −5.2 kcal mol^−1^ and established polar interactions with residues Y68, Y122, H374, and S375 (Figure 13).

**Figure 14:**
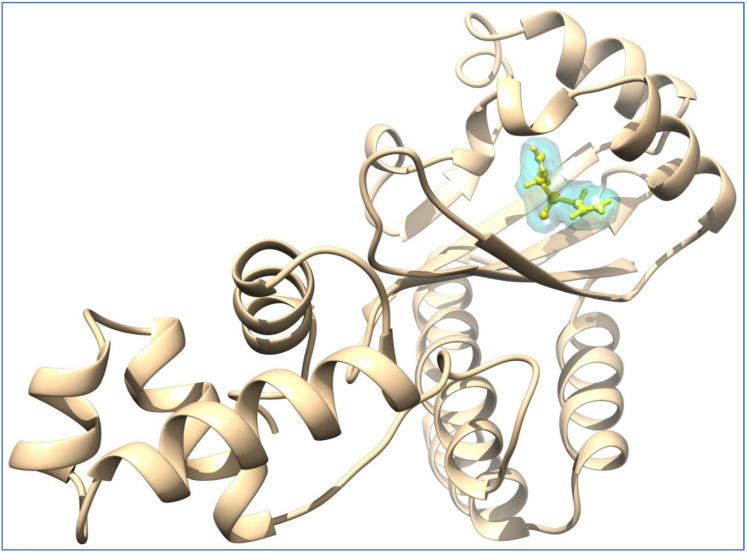
Best-scoring docking pose of allicin in SidA. Allicin (yellow, ball-and-stick) is embedded in the SidA binding pocket (shown as blue surface style); the protein is depicted as a solid ribbon. Adapted from Yazdani et al. [228].

Yazdani et al. conducted docking studies of allicin within the ligand-binding pocket of the SidA quorum-sensing receptor [228]. The highest-ranked pose exhibited a predicted affinity of −5.1 kJ mol^−1^, suggesting a favorable binding profile (Figure 14). The model includes hydrogen bonds between the sulfur center of allicin and residues S39 and S130, as well as an electrostatic interaction with D76. The combination of bonding and non-bonding interactions explains the accommodation of allicin within SidA’s pocket and may facilitate competitive engagement of the receptor site.

### 4.2. Molecular Dynamics Simulation

Molecular dynamics (MD) simulation is integral to structure-guided investigations of garlic phytochemicals, as it effectively captures protein-ligand flexibility, solvent interactions, and the durability of critical contacts beyond static docking. This capability enhances mechanistic hypotheses and facilitates the prioritization of leads before experimental validation [229,230,231,232]. In various disease areas, MD has been employed to analyze canonical stability descriptors, including root-mean-square deviation (RMSD) and root-mean-square fluctuation (RMSF) of protein backbones, hydrogen-bond duration, radius of gyration (Rg), and solvent-accessible surface area (SASA) for OSCs like allicin, alliin, and related sulfides [233].

In oncology pipelines, garlic-derived candidates identified through docking against NSCLC targets (EGFR, HER2, EML4-ALK) were refined using MD to confirm pose robustness and interaction networks, demonstrating that dynamics enhance the reliability of virtual screening results [234]. MD is also used in neuroprotective applications. A 100 ns study demonstrated that alliin and allicin form structurally stable complexes with α-synuclein, with MM-GBSA energy computations, supporting alliin and *in vitro* validation in SH-SY5Y cells confirming anti-aggregation effects [235].

In antiviral research, MD has confirmed docking-derived poses for garlic constituents at the SARS-CoV-2 main protease (Mpro), specifically *γ*-glutamyl-*S*-allylcysteine and SAC, and has also been utilized for complexes involving the spike/ACE2 interface [236,237]. In addition to virology, toxicology research integrated docking with MD to elucidate allicin’s binding mode in CYP2E1, correlating the predicted stable complex with diminished reactive oxygen species values *in vitro* [238]. Furthermore, antifungal workflows have integrated reverse docking with MD to assess the stability of garlic metabolites in relation to fungal CYP51, transitioning from score-based ranking to time-resolved interaction analyses [227]. MD has elucidated host-directed mechanisms, with simulations indicating that *S*-1-propenyl-*L*-cysteine engages TLR4, aligning with the observed anti-inflammatory signaling [239]. Recent computational and experimental analyses of topoisomerase IV employed MD, utilizing RMSD, Rg, SASA, and hydrogen bond tracking to demonstrate stable allicin-enzyme complexes and coordinated motions, thereby reinforcing the proposed antibacterial mechanisms of garlic OSCs [224].

### 4.3. Network Pharmacology

Network pharmacology research consistently illustrates that allicin and its derivatives function as multi-target agents influencing lipid metabolism, inflammation, oxidative stress, and apoptotic pathways. In palmitate-induced steatosis, allicin was associated with PPAR signaling nodes and confirmed by qPCR to up-regulate PPARA and FABP6 while down-regulating FABP4 and PPARG, demonstrating a compound-target-pathway framework anchored in the mechanism [240]. Alliin has been identified as a multitarget autophagy modulator through the integration of target prediction, PPI-network analysis, GO/KEGG enrichment, docking, and experimental results. In addition to its role in metabolic regulation, the phytochemistry of garlic has been examined in relation to complex diseases [241]. Network pharmacology, along with docking and experimental validation, suggests that garlic compounds may play a role in alleviating alcoholic liver disease and atherosclerosis [242], particularly through ferroptosis-related pathways [225,243]. Recent studies in oncology have identified allicin as a potential therapeutic agent for non-small cell lung cancer [244]. Phenotypic anti-infective effects correspond with these networks; for instance, DADS inhibits quorum sensing, virulence factors, and biofilm formation in *Pseudomonas aeruginosa*, supporting broader evidence that garlic organosulfurs function as bactericidal, antibiofilm, anti-toxin, and anti-QS agents against various pathogens [40,245,246].

### 4.4. Quantitative Structure–Activity Relationships (QSAR)

QSAR modeling of allicin analogs utilizes topological, electronic, and lipophilicity descriptors to elucidate antiviral potencies and prioritize substitutions, while docking offers complementary target-binding hypotheses. Structure-activity analyses of allicin-derived disulfides for antibacterial applications reveal coherent SAR trends, indicating that heteroaryl or quinazolinyl disulfides enhance both potency and stability [247,248]. These studies frequently support thiol-reactive mechanisms via thiol-disulfide exchange. Chain-length effects in allyl polysulfides elucidate activity, where an increase in sulfur atoms (DAS < DADS < DATS and higher) is typically linked to a stronger biological response, aligning with improved thiol-modifying ability and hydrogen-sulfide–releasing potential [249]. QSAR and SAR identify substituent patterns, sulfur chain length, and electrophilicity as critical factors for enhancing potency, selectivity, and developability in antimicrobial, metabolic, cardiovascular, and anticancer applications [250,251].

### 4.5. Density Functional Theory Computations

**Figure 15:**
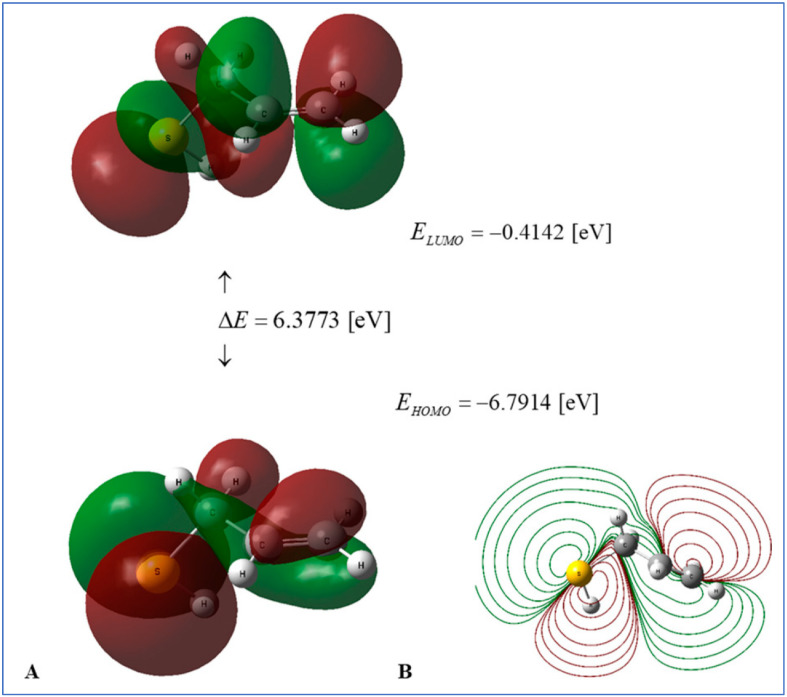
Frontier orbitals and electrostatic potential of allyl mercaptan. (**A**) HOMO–LUMO energy levels and isosurfaces in water (DFT B3LYP/cc-pVQZ, C-PCM). (**B**) Molecular electrostatic potential (MEP) contoured on the electron-density surface. Adapted from Molski 2024 [25].

Recent studies employing density functional theory (DFT) have elucidated the roles of essential OSCs in garlic as antioxidants, redox modulators, and reaction intermediates. In this regard, Molski assessed thermodynamic and global reactivity descriptors for 2-propenesulfenic acid and allyl mercaptan using B3LYP/cc-pVQZ with implicit water [25]. The findings indicate that in aqueous environments, the sequential proton loss-electron transfer pathway is preferred, whereas hydrogen atom transfer in the gas phase may prevail. These results elucidate the enhanced radical-trapping ability of sulfenic acids derived from allicin (Figure 15). In addition to intrinsic reactivity, DFT has played an important role in delineating chemically significant transformations of garlic polysulfides. Cai and Hu described the hydrogen sulfide-releasing mechanism of diallyl di- and trisulfide through thiolate attack, elucidating the greater H_2_S donation capacity of diallyl trisulfide compared to the disulfide [252].

DFT study was used to elucidate the gas-phase dehydration mechanisms of the allicin radical cation compared to its protonated form, corroborating experimentally observed pathway preferences [253]. Furthermore, recent multiscale DFT studies of allicin-nanocage complexes indicate increased stability, reactivity, and target engagement, proposing formulation strategies that could enhance bioavailability [254]. Likewise, DFT has been employed to elucidate the geometry and isomer preference in metal complexes of *S*-allyl-*L*-cysteine, establishing a connection between structure and antibacterial activity [255].

Arroio et al. employed density functional theory to compute and correlate essential electronic descriptors of ajoene with its proposed antioxidant properties [256]. The findings indicate that the E-isomer is thermodynamically preferred compared to the Z-isomer and demonstrates significant electron-accepting properties. The electrophilic nature and ability for charge-transfer interactions establish a mechanistic foundation for the redox activity of E-ajoene, indicating its potential to interact with biological targets via donor–acceptor pathways [257].

Benchmarking of S–S bond descriptions indicates that disulfide-containing systems, such as thiosulfinates and disulfides, necessitate the selection of appropriate modern functionals to ensure accurate structural and vibrational properties [258]. In conjunction with kinetic DFT studies indicating that 2-propenesulfenic acid exhibits significantly greater reactivity toward peroxyl radicals compared to allicin, these results establish DFT as a pivotal instrument for the mechanistic understanding and development of garlic-derived pharmaceuticals [259].

### 4.6. ADMET Studies

Computational ADME/Tox screening is routinely utilized for garlic OSCs to prioritize candidates prior to laboratory assessments. Standard workflows integrate property and liability prediction tools, including SwissADME [260], pkCSM [261], admetSAR 2.0 [262], and ADMETlab 2.0 [263], as well as toxicity classifiers such as ProTox-II [264]. These tools are utilized to estimate gastrointestinal absorption, blood–brain barrier (BBB) penetration, P-glycoprotein interaction, and potential cytochrome P450 inhibition. The studies of garlic indicate significant predicted gastrointestinal absorption for various organosulfurs, with allicin and allyl sulfides (DAS, DADS, DATS) showing potential for BBB penetration [265]. In contrast, more polar compounds, such as SAC, exhibit reduced likelihood of crossing the BBB. Permeability modeling integrating immobilized artificial membrane (IAM) chromatography with chemometric/QSAR analysis indicates a strong correlation between hydrophobic descriptors (e.g., log k′(IAM)) and predicted human intestinal absorption, BBB permeation, and skin permeability, which illustrates the importance of lipophilicity in this chemotype [266]. Recent benchmarking of ADMET tools highlights variability across endpoints and suggests that the selection of tools and interpretation of endpoints should be aligned with emerging experimental data [267].

## 5. Safety Considerations and Drug Interactions

Safety assessments of garlic’s primary OSCs suggest a largely favorable profile at culinary or standard supplemental levels; however, significant risks arise with concentrated formulations, elevated dosages, parenteral administration, inappropriate topical use, or particular drug interactions [40,268,269]. Allicin exhibits chemical reactivity and potential mucosal irritation. Recent reviews provide a summary of its absorption, metabolism, and dose-dependent adverse effects, including gastrointestinal disturbances and contact sensitivity, along with the preparation type and stability [270]. Regulatory and expert panels evaluating allyl sulfides as flavoring substances typically identify no safety concerns with reported dietary intakes, while also noting data gaps for specific members of the class [271,272].

Recent toxicological research on DATS indicates that prolonged high-dose administration may present risks. A report of Wu et al., which compiled acute and subacute studies, proposed a conservative upper intake estimate for adults of approximately 359 mg of DATS per day (equivalent to about 84.5 g of garlic), emphasizing that apoptosis-related mechanisms are associated with supradietary exposure [273]. Topical application of raw garlic is associated with various cutaneous injuries, ranging from irritant or allergic contact dermatitis to second-degree burns. A systematic review has compiled numerous cases of burns, and recent reports highlight incidents of infant injuries resulting from garlic poultices [268,274]. Hypersensitivity reactions vary from fingertip pulpitis and occupational asthma to rare instances of anaphylaxis [275,276].

Diallyl disulfide is identified as a contact allergen, with contemporary reviews highlighting that raw handling poses a greater risk than exposure to cooked forms. Garlic derivatives, particularly ajoene, have demonstrated both mechanistic and experimental evidence for platelet inhibition. However, dietary garlic does not significantly affect platelet function in healthy individuals. Despite this, anesthesia societies advise discontinuing herbal supplements, including garlic, one to two weeks prior to surgery to reduce the risk of bleeding complications [269,277,278,279].

Clinically relevant drug interactions are a critical safety concern. Aged garlic extract did not elevate haemorrhagic events in patients monitored while on warfarin [280]. However, a controlled crossover trial indicated that garlic capsules significantly decreased plasma saquinavir concentrations, highlighting potential impacts on xenobiotic metabolism and the necessity for individual assessments with narrow-therapeutic-index medications [281]. Moreover, hepatic adverse events are infrequent but may occur, as evidenced by case reports of reversible hepatotoxicity following high-dose commercial garlic products. Animal studies suggest liver injury at significantly elevated daily intakes, necessitating the avoidance of excessive doses and careful monitoring in individuals with liver disease [282,283].

## 6. Future Perspectives

The future prospects of garlic as a therapeutic agent signify a transition from conventional folk medicine to advanced, evidence-based therapeutic strategies that utilize modern biotechnology and precision medicine techniques. Recent studies indicate that garlic is evolving into next-generation therapeutics via advanced nanotechnology-based drug delivery systems, such as silver nanoparticle conjugates and microencapsulation technologies, which improve bioavailability and targeted therapeutic efficacy. The incorporation of artificial intelligence in computer-aided drug design is transforming garlic-based drug discovery. Computational models are identifying optimal bioactive compounds and predicting therapeutic interactions for significant health issues, including COVID-19, antimicrobial resistance, and cancer. Future applications are expected to include personalized medicine strategies that exploit pharmacogenomics to enhance garlic-derived treatments according to individual genetic profiles. Additionally, organ-on-chip technologies offer novel platforms for evaluating garlic therapeutics in physiologically relevant human tissue models. The integration of garlic research with microbiome science, immunomodulation, and precision nutrition is advancing these historical therapeutic agents within modern healthcare. Current clinical trials are investigating their roles in optimizing gut health, protecting cardiovascular function, and enhancing immune system performance through evidence-based, bioengineered formulations that preserve traditional efficacy while adhering to contemporary pharmaceutical standards.

## 7. Conclusions

Garlic demonstrates consistent effectiveness in treating cardiovascular and metabolic disorders, notably in lowering blood pressure, enhancing lipid profiles, and regulating inflammatory markers. Clinical trials and meta-analyses provide evidence for its supplementary role in the management of hypertension, dyslipidemia, and elements of metabolic syndrome. Recent data indicate potential neuroprotective effects, particularly in Alzheimer’s and other neurodegenerative models, as well as significant anticancer properties via multi-targeted apoptosis induction and cell cycle regulation. Its antimicrobial properties contribute to its extensive range of therapeutic applications. Clinical validation for neurodegenerative and oncological indications is constrained by small sample sizes, variability in garlic preparations, and a lack of large-scale randomized controlled trials.

In addition to basic preparations, various formulations seek to standardize dosage and enhance the bioavailability of garlic’s OSCs. Aged garlic extract, stabilized allicin products, and garlic oil are delivered as enteric-coated tablets, capsules, or odor-masked liquids to limit gastric degradation and enhance intestinal release; newer systems such as nanoemulsions, cyclodextrin inclusions, liposomes, solid-lipid nanoparticles, polymeric nanoparticles and pH-responsive matrices, hydrogels, films, ethosomes, and niosomes seek improved solubility, permeability, and sustained release. Simultaneously, computational studies are progressively informing target selection and scaffold optimization: docking and molecular dynamics elucidate multi-target engagement, while DFT analyses of HOMO–LUMO gaps, electrophilicity, and sulfur redox states clarify the covalent and non-covalent reactivity of allicin, ajoene, alliin, and allyl mercaptan. QSAR, network pharmacology, and *in silico* ADMET screenings are utilized to prioritize leads and formulation strategies prior to *in vitro* validation. Taken together, pharmacological evidence, formulation innovations, and computational workflows are collectively guiding the field toward reproducible, mechanism-based garlic therapeutics. Nevertheless, standardized preparation characterization and prospective pharmacokinetic–pharmacodynamic studies are essential for clinical translation.

## Figures and Tables

**Table 1:** An overview of the molecular targets and mechanisms of action of garlic organosulfur compounds, and the key findings from relevant studies.

S.No.	Molecular Targets/Pathway	Study Type	Main Findings	References
1.	Keap1/Nrf2/ARE signaling	*In vitro* (SH-SY5Y Cell)	Curcuma- and garlic-derived hybrids exhibited antioxidant action by stimulating the Nrf2 signaling pathway and increasing ARE-regulated expression of downstream target genes, such as heme oxygenase-1 (HO-1) and NQO1, while having no effect on miR-125b-5p microRNA expression.	[154]
		*In vivo* (LPS-induced acute lung injury)	Garlic-derived SAMC (10, 30, or 60 mg/kg) reversed lipopolysaccharide-induced lung injury in BALB/c mice by activating the Keap1/Nrf2 pathway, which increased HO-1 and NQO1 expression, suppressed pro-inflammatory cytokines (TNF-α, IL-1β, IL-6, iNOS, and COX2), and inhibited the NF-κB signaling pathway.	[155]
		*In vivo* (DSS-induced IBS and SLI)	Garlic polysaccharides (PSG) reduced secondary liver injury (SLI) and inflammatory bowel disease (IBD) in mice. PSG suppressed pyroptosis by downregulating IL-1β, IL-18, NLRP3, gasdermin D, caspase-1, ASC, TLR4, MyD88, NF-κB, and phospho-NF-κB, increased anti-inflammatory responses through IL-10 production, and alleviated oxidative stress by activating the Keap1/Nrf2/ARE signaling pathway.	[156]
		*In vitro* (Human umbilical vein endothelial cells)	Aged garlic extract (AGE) enhances cellular antioxidant defenses by activating the Nrf2-ARE pathway, leading to increased expression of HO-1 and glutamate-cysteine ligase modifier subunit (GCLM) in human umbilical vein endothelial cells.	[157]
2.	Cyclooxygenase (COX2) and lipoxygenase (LOX)	*In vivo* (Paw edema in rats) & *In vitro* (RAW264.7 cells)	Diallyl trisulfide, an OSC derived from garlic, inhibited cyclooxygenase and inducible nitric oxide synthase by suppressing the AKT1/TGF-β-driven activation of the MAPK/NF-κB signaling pathway in LPS-induced paw edema in rats and RAW264.7 macrophage cells.	[158]
		*In vitro* (RAW264.7 macrophage cells)	Garlic bioactive constituents, such as allicin and Z-ajoene, inhibited the phosphorylation and nuclear translocation of Signal Transducer and Activator of Transcription 3 (STAT3) and suppressed COX2 activity in RAW264.7 murine macrophages.	[159]
		*In vitro*	Diallyl monosulfide and diallyl disulfide inhibit LOX by creating an adsorption embolism within the enzyme’s substrate channel, whereas diallyl trisulfide, and allicin disrupt its function by interfering with interactions between the N- and C-terminal domains.	[160]
		*In vivo* (Rabbits platelets, lung, and aorta)	Raw garlic extract inhibited COX activity more effectively than cooked garlic extract, showing a stronger effect on platelet COX activity.	[74]
		*In vitro* (RAW264.7 cells)	Aged black garlic extract showed greater antioxidant activity in ABTS and DPPH radical scavenging assays but exhibited weaker anti-inflammatory effects, as revealed by reduced inhibition of cyclooxygenase-2 and 5-lipoxygenase, compared with fresh raw garlic extract in LPS-activated RAW264.7 cells.	[161]
		*In silico* (Molecular docking and dynamics simulation)	Alliin exhibited the greatest binding affinity and improved stability in the COX-2 active site as compared to celecoxib using the MM/PBSA methods.	[162]
3.	eNOS/NO pathway	*In vitro* (H9c2 myoblast cells)	Allicin protects H9c2 cells from ischemic hypoxia-induced apoptosis by stimulating the eNOS/NO pathway and exerting antioxidant effects via increased expression of Nrf2 and heme oxygenase-1 (HO-1) proteins.	[163]
		*In vitro* (EA.hy 926 cells)	Endothelial cells treated with aged garlic extract (5 mg/mL) prevented HAT/MET-induced endothelial dysfunction by restoring nitric oxide levels and preserving tetrahydrobiopterin from oxidative damage, even in high-Hcy environments.	[164]
		*In vitro* (Mouse macrophage cell line)	Garlic extract and SAC increased NO in endothelial cells by preventing the activation of NF-κB signaling in LPS and IFNγ-stimulated RAW264.7 cells, while also reducing the generation of hydroxyl free radicals and controlling NO production by blocking iNOS expression in macrophages.	[165]
		*In vitro* (Platelets and human placental villous tissue)	Garlic extracts in a 1% alcohol solution induced concentration-dependent nitric oxide (NO) production by activating calcium-dependent nitric oxide synthase in both platelets and human placental villous tissue.	[166]
		*In vitro* (Aortic rings of rat)	Aged garlic extract produced dose-dependent vasorelaxation of the aortic endothelium by increasing NO generation in norepinephrine-pretreated cells, which was reversed by NOS inhibitors and NO scavengers.	[167]
4.	PPAR-γ/NF-κB signaling	*In vitro* (Aortic smooth muscle cells, A7r5)	Garlic-derived diallyl sulfide inhibited ROS-driven PI3K/Akt signaling by stimulating Nrf2 pathway and downstream NF-κB and AP-1 activation through the dissociation of TRADD and TRAF2 in rat A7r5 cells.	[168]
		*In vivo* (DSS-induced colitis in mice) & *In vitro* (LPS-stimulated RAW264.7 cells)	Alliin effectively attenuates inflammation in both DSS-induced colitis in mice and LPS-stimulated RAW264.7 macrophages by inhibiting MAPK signaling, suppressing PPAR-γ phosphorylation, and blocking the downstream activation of AP-1, NF-κB, and STAT-1.	[169]
		*In vivo* (LPS-induced ALI)	Alliin mitigates LPS-induced lung inflammation and injury in BALB/c mice by activating PPARγ and inhibiting NF-κB signaling.	[170]
		*In vivo* (LAD-induced MI in C57BL/6 mice) & *In vitro* (Isolated cardiomyocyte)	Alliin (100 mg/kg, intraperitoneal) protected C57BL/6 mice from LAD-induced myocardial infarctions. It inhibited necroptosis by reducing the expression of RIP1, RIP3, and TRAF2, while boosting autophagy through elevation of PPARγ expression in hypoxia-induced necroptosis in H9C2 cells.	[171]
5.	MAPK–CREB–BDNF axis	*In vitro* (6-OHDA treated SH-SY5Y cells) & *In silico* (Network pharmacology and molecular docking)	Allicin (ALC; 10, 50, and 100 μM) reduced 6-OHDA-induced cytotoxicity in SH-SY5Y cells by increasing dopamine transporter (DAT) levels and Bcl-2 expression, while decreasing Bax expression, primarily through activation of the PKA/p-CREB/BDNF signaling pathway. *In silico* analysis further revealed that ALC exerts its effects via 19 molecular targets, including components of the PI3K-Akt, MAPK, and cAMP signaling pathways.	[26]
		*In vitro* (NP cells) & *In vivo* (C57BL/6 mice)	Diallyl disulfide (DADS; 10 mg/kg) may negatively influence hippocampal neurogenesis and cognitive function by disrupting ERK and BDNF-CREB signaling. It also showed reduced proliferation neural progenitor cells (NPC) in the dentate gyrus.	[172]
		*In vitro* (Cortical slices of rats)	SAC (100 μM) ameliorates quinolinic acid–induced excitotoxic oxidative damage in isolated rat cortical slices through its anti-inflammatory and antioxidant effects, mediated by activation of the Nrf2/ARE pathway, which suppresses TNF-α and increases BDNF levels.	[173]
6.	Tissue remodeling factors (MMP-9)	*In vitro* (Chondrocyte model of OA)	In an IL-1β-induced OA model, SAMC from garlic exhibits anti-inflammatory and chondroprotective effects by suppressing NF-κB signaling, restoring cartilage matrix balance (through reduced MMP activity and increased TIMP-1 expression), and lowering TNF-α mRNA expression in cell supernatants.	[174]
		*In vitro* (EJ bladder cancer cells)	Garlic extract exerts potent anti-tumor effects on EJ bladder carcinoma cells by inducing cell cycle arrest, modulating key signaling pathways, and suppressing invasion-related transcriptional activity, including the downregulation of MMP-9 and reduced binding activities of AP-1, Sp-1, and NF-κB, ultimately leading to decreased tumor invasion.	[175]
7.	P53 gene	*In vivo* (DENA and 2-AAF-induced hepatic cancer in rats)	Garlic oil pretreatment partially corrected the architectural changes induced by diethylnitrosamine (DENA) and 2-acetylaminofluorene (2-AAF) in rat hepatic cancer through its antioxidant effects and by suppressing p53 gene expression.	[176]
		*In vitro* (MCF-7 and MD-MBA-231 cells)	Allicin reduces cell viability and induces apoptosis and cell cycle arrest in breast cancer cells by boosting the mRNA expression of A1BG and THBS1 while suppressing the expression of TPM4 via p53 activation.	[177]
		*In vitro* (Human AGS cells)	The polyphenolic extract of lyophilized garlic displayed a dose-dependent anticancer impact via raising p53 expression, resulting in a rise in the pro-apoptotic protein (Bax activity), a decrease in the anti-apoptotic protein (Bcl-2), and suppression of the PI3K/Akt pathway.	[178]
8.	Growth factors (EGFR)	*In vitro* (Colorectal cancer cells) & *In silico* (Discovery Studio 4.0)	Alliin, *S*-allyl-*L*-cysteine-sulfoxide derivative from garlic demonstrated an inhibitory effect on Epidermal Growth Factor Receptor (EGFR), attributed to its higher binding affinity, which led to a reduction in the viability of colorectal cancer cells.	[179]
		*In vivo* (Erlotinib-induced skin toxicity in mice)	Diallyl trisulfide (DATS) alleviates EGFR inhibitor (erlotinib)-induced skin toxicities by suppressing inflammatory cytokines such as TNF-α and IL-6, which in turn reduces follicle count, keratin hyperplasia, and neutrophil infiltration in mice.	[180]
9.	Intrinsic (mitochondrial) apoptotic pathway	*In vitro* (Gastric cancer cells)	Allicin decreased the viability of SGC-7901 gastric cancer cells in a dose- and time-dependent fashion by triggering apoptosis through mitochondrial cytochrome c release, activating caspases-3, -8, and -9, and enhancing Bax and Fas expression via modulating both intrinsic and extrinsic apoptotic pathways.	[181]
		*In vitro* (Human cancer cells and rat liver mitochondria)	Aged garlic extract (AGE) and *S*-allyl-*L*-cysteine (SAC) induce cytotoxicity in tumor cells by triggering mitochondrial permeability transition (MPT) in human cancer cell lines and in rat liver mitochondria.	[182]
		*In vitro* (ATC cells)	Diallyl sulfide (DAS) inhibits growth and triggers apoptosis in anaplastic thyroid carcinoma (ATC) cells by downregulating Bcl-2 and upregulating Bax, promoting cytochrome c release, activating caspase-9 and caspase-3, and cleaving PARP via the mitochondrial signaling pathway.	[183]
10.	PI3K/Akt pathway	*In vivo* (Fructose fed diabetic rats)	Raw garlic homogenate (250 mg/kg/day) reverses NF-κB activity and reduces cardiac oxidative stress markers (catalase, GPx, and GSH) by activating the PI3K/Akt/Nrf2 pathway and downregulating Keap1 levels in fructose-induced diabetic rats.	[184]
		*In vitro* (Lung adenocarcinoma cells)	Allicin suppresses lung adenocarcinoma cell adhesion, migration, and invasion in a dose-dependent manner by modulating MMP/TIMP expression and inhibiting PI3K/AKT signaling, primarily through the suppression of AKT phosphorylation without affecting AKT protein expression.	[185]
11.	Wnt/β-catenin	*In vivo* (mice) & *In vitro* (GE1 cells)	Aged garlic extract (2 g/kg/day) inhibited the progression of periodontal disease in rats by upregulating Defb4 mRNA expression in gingival tissue via activation of the Wnt/β-catenin signaling pathway.	[186]
		*In vitro* (Gastric carcinoma cells)	Diallyl disulfide (DADS) inhibits the proliferation, transition, migration, and invasion of gastric carcinoma cells by downregulating the expression of target genes such as Axin, c-Jun, and c-Myc through the Wnt/β-catenin signaling pathway, while simultaneously enhancing the interaction between RORα and β-catenin.	[187]
		*In vitro* (Breast cancer stem cells)	The suppression of lithium chloride-induced Wnt/β-catenin signaling activation by diallyl trisulfide (DATS) resulted in the reduction in the expression of CSC markers (CD44, ALDH1A1, Nanog, and Oct4) and the formation of tumor spheres in breast cancer stem cells.	[188]
12.	Cyclin/CDK	*In vitro* (Chondrocyte cells)	Allicin promoted chondrocyte proliferation by upregulating the expression of cyclin D1, CDK4, and CDK6 at both the mRNA and protein levels, facilitating the G1-to-S phase transition of the cell cycle, and increasing cell viability in a dose-dependent manner.	[189]
		*In vitro* (U937 cells)	N-benzyl-N-methyldecan-1-amine (NBNMA), a phenylamine derivative of garlic, exhibits potent anticancer activity by restricting cell growth and cell cycle progression, as well as by inducing apoptosis through caspase-3 activation, PARP destruction, an elevated Bax/Bad to Bcl-2/Bcl-xL ratio, and the inhibition of mitochondrial proteins XIAP and cIAP-1.	[190]

Abbreviations: 2-AAF, 2-acetylaminofluorene; A1BG, Alpha-1-B glycoprotein; ABTS, 2,2′-azino-bis(3-ethylbenzothiazoline-6-sulfonic acid); AGE, Aged garlic extract; AGS, Gastric adenocarcinoma cells; Akt, Alpha serine/threonine-protein kinase; ALDH1A1, Aldehyde Dehydrogenase 1 Family Member A1; AP-1, Activator protein 1; ARE, antioxidant response element; ASC, Apoptosis-associated speck-like protein containing a CARD; Bad, BCL-2 antagonist of cell death; Bax, Bcl-2 associated X protein; Bcl-2, B-cell lymphoma-2; BDNF, Brain-Derived Neurotrophic Factor; cAMP, Cyclic adenosine monophosphate; CD44, Cluster of differentiation 44; cIAP-1, Cellular inhibitor of apoptosis protein-1; c-Myc, Cellular myelocytomatosis oncogene; COX2, Cyclooxygenase-2; CSC, Cancer stem cell; CDK4 and CDK6, Cyclin-Dependent Kinase 4 and 6; DADS, Diallyl disulfide; DAT, Dopamine transporter; DATS, Diallyl trisulfide; DENA, Diethylnitrosamine; DPPH, 2,2-Diphenyl-1-picrylhydrazyl; DSS, Dextran sodium sulfate; EGFR, Epidermal Growth Factor Receptor; eNOS, Endothelial nitric oxide synthase; ERK, Extracellular signal-regulated kinase; GCLM, Glutamate-cysteine ligase modifier subunit; GPx, Glutathione peroxidase; GSH, Glutathione; HAT/MET, Heat stress and methionine overload; Hcy, Homocysteine; HO-1, Heme oxygenase-1; IBD, Inflammatory bowel disease; IL-6/18, Interleukin-6/18; iNOS, inducible nitric oxide synthase; Keap1, Kelch-like ECH-associated protein 1; LAD, Left anterior descending (LAD) artery; LOX, Lipoxygenase; LPS, Lipopolysaccharide; MAPK, Mitogen-activated protein kinases; MCF-7, Michigan cancer foundation-7 (Breast cancer cell line); MI, Myocardial infarction; MMP, Matrix metalloproteinase; MPT, Mitochondrial permeability transition; MyD88, Myeloid differentiation factor 88; NBNMA, N-benzyl-N-methyldecan-1-amine; NF-kB, Nuclear factor kappa-light-chain-enhancer of activated B cells; NLRP3, Nucleotide-Binding Oligomerization Domain, Leucine-Rich Repeat and Pyrin Domain-Containing 3; NO, Nitric oxide; NPC, Neural progenitor cells; NQO1, NAD(P)H:quinone oxidoreductase 1; Nrf2, Nuclear factor-erythroid 2-related factor 2; OA, Osteoarthritis; Oct4, Octamer-binding transcription factor 4; p-CREB, phosphorylated cAMP-responsive element-binding protein; PI3K, Phosphatidylinositide 3-kinases; PKA, Protein kinase A; PPARγ, Peroxisome-proliferator-activated receptor γ; PSG, Polysaccharide; RIP1/3, Receptor Interacting Protein 1/3; RORα, Retinoic acid-related orphan receptor alpha; ROS, reactive oxygen species; SAC, *S*-allylcysteine; SAMC, *S*-allylmercaptocysteine; Sp-1, Specificity protein 1; STAT-1, Signal Transducer and Activator of Transcription 1; STAT3, Signal transducer and activator of transcription 3; THBS1, Thrombospondin-1; TIMP-1, Tissue Inhibitor of Metalloproteinase-1; TLR4, Toll-like receptor 4; TNFα, Tumor necrosis factor alpha; TPM4, Tumor progression and metabolism 4; TRADD, TNF receptor-associated death domain protein; TRAF2, Tumor necrosis factor receptor-associated factor 2; XIAP, X-linked inhibitor of apoptosis protein.

## Data Availability

No data was used for the research described in the article.
